# Urinary Metabolomic Profiling Analysis and Evaluation of the Effect of *Ecklonia cava* Extract Intake

**DOI:** 10.3390/nu12051407

**Published:** 2020-05-14

**Authors:** Juyeon Kim, Youngae Jung, Eunok Lee, Seoyeong Jang, Do Hyun Ryu, Oran Kwon, Geum-Sook Hwang

**Affiliations:** 1 Integrated Metabolomics Research Group, Western Seoul Center, Korea Basic Science Institute, Seoul 120-140, Korea; juyeon9014@naver.com (J.K.); jya0819@kbsi.re.kr (Y.J.); jm0326@kbsi.re.kr (S.J.); 2 Department of Chemistry, Sungkyunkwan University, Suwon 440-746, Korea; dhryu@skku.edu; 3 Department of Nutritional Science and Food Management, Ewha Womans University, Seoul 03760, Korea; flffl0204@naver.com; 4 Korea Basic Science Institute, 150, Bugahyeon-ro, Seodaemun-gu, Seoul 03759, Korea

**Keywords:** *Ecklonia cava*, seapolynol, metabolomics, antioxidant effect, mass spectrometry

## Abstract

Metabolomics is a powerful tool for the investigation of interactions between diet, nutrients, and human metabolism. *Ecklonia cava* is an edible brown alga that is abundantly found in Korea and Japan and contains unique polyphenols referred to as phlorotannins. However, there are few metabolomics studies related to the effects of polyphenols in humans. In this study, we performed a mass spectrometry-based metabolomics analysis of urine samples from participants with a body mass index (BMI) higher than 25 kg/m^2^ and lower than 30 kg/m^2^ to investigate the effects of the intake of seapolynol isolated from *E. cava*. Metabolomic profiling showed that the levels of riboflavin, urocanic acid, 5-hydroxy-6-methoxyindole glucuronide, and guanidino valeric acid were significantly increased in the seapolynol intake group compared with the placebo group. A correlation analysis was performed to identify the association between the metabolites’ levels and clinical characteristics related to body fat. Among the metabolites whose concentrations changed in the seapolynol intake group, riboflavin was associated with BMI, body weight, fat mass, and percent body fat. These findings suggest that the decreased body fat induced by the intake of seapolynol is related to an increase in the antioxidant effect of riboflavin.

## 1. Introduction

*Ecklonia cava* is an edible brown alga that is not available in Europe but is abundantly found in Korea (30,000 t per year) and Japan [1]. It has already been used in various fields, for instance, as a food ingredient, in medicine, and in animal feeds. Phlorotannins, including dieckol, eckol, bieckol derivatives, phlorofurofucoeckol, and phloroeckol, are abundant in *E. cava* and are considered major antioxidant components due to their action against free radicals [1,2]. Recent research found that *E. cava* extracts can affect body fat and body weight in humans [3]. Lee et al. reported that abdominal fat was significantly reduced in obese Korean people by the intake of *E. cava* extracts [4].

The incidence of obesity has steadily increased over the last three decades worldwide [5]. Obesity is a complex disease that is affected by genetic factors, eating habits, and several environmental factors. In particular, excessive caloric intake and low consumption of calories contribute to energy imbalance, which can easily lead to obesity [6,7]. Although proper exercise and diet are the most basic ways to control obesity, the use of drugs and the management of the effects of their possible overdose are more convenient for modern people. However, due to various side effects, the intake of functional foods instead of drugs has begun to gain popularity [8].

Metabolomics is a powerful tool to study the interactions between diet, nutrients, and human metabolism [9,10,11,12]. Nutritional intervention studies that used metabolomics have characterized the response to foods in humans [13,14,15] as well as animal [16,17,18] models and have investigated the effects of different diets [19,20].

Previous studies mainly reported the effects and/or biological functions of *E. cava* extracts. However, few studies have examined the metabolic changes after intake of *E. cava* extracts. Here, we investigated the metabolic effects of a 12-week consumption of *E. cava* polyphenol extracts (seapolynol) in subjects exposed to moderate caloric intake and physical activity and with a body mass index (BMI) higher than 25 kg/m^2^ and lower than 30 kg/m^2^.

## 2. Materials and Methods

### 2.1. Subjects and Seapolynol Supplement

The design of this randomized, double-blind, parallel study has been described previously [4]. In brief, 56 overweight healthy subjects [mean ± SD age, 36.8 ± 1.2 years; body mass index, 27.4 ± 0.2 kg/m^2^] consumed a placebo or a seapolynol supplement containing polyphenol (4 capsules per day) for a 12-week period. There were no significant adverse symptoms reported. All participants gave written informed consent before enrolment. The study was approved by the Institutional Review Boards of Ewha Womans University (IRB NO. 67-14) and was registered in the International Clinical Trials Registry Platform of the WHO (No. KCT0000276). The Consolidated Standards of Reporting Trials (CONSORT) flow diagram was used to ensure that the study was carried out according to established guidelines.

### 2.2. Urine Sample Collection

Urine samples were collected from 56 subjects after an overnight fast of ≥12 h before seapolynol supplementation and at 12 weeks after seapolynol supplementation. The urine samples were stored in a freezer at −80 °C until analysis.

### 2.3. Materials and Chemicals

All solvents, including water, used for ultra-performance liquid chromatography/quadrupole time-of-flight mass spectrometry (UPLC/QTOF MS) analysis were of LC–MS grade and purchased from Scharlau (Scharlau, Sentmenat, Spain). The formic acid used in binary solvents was obtained from TCI (TCI, Tokyo, Japan). Other standards used to confirm the metabolites, namely, riboflavin, urocanic acid, 5-methoxyindole, and guanidine, were analytical-grade and obtained from Sigma-Aldrich (Sigma-Aldrich Chemie Gmbh, Steinheim, Germany). Valeric acid was obtained from Fluka (Honeywell Fluka™, Fluka, Germany).

### 2.4. Sample Preparation

Two hundred microliters of urine samples were thawed and vortexed for 1 min. Then, 1 mL of acetonitrile (ACN) was added to each urine sample to precipitate proteins, and the mixture was centrifuged for 10 min at 13,000 rpm and 4 °C. The aqueous supernatant was transferred to a new 1.5 mL Eppendorf tube and dried in a vacuum concentrator for 2 h. The samples were dissolved in 1.4 mL of 20% ACN for LC–MS analysis. The dissolved samples were centrifuged for 10 min at 13,000 rpm and 4 °C before being transferred to inlet vials.

### 2.5. Metabolomic Analysis Using LC–MS

For global profiling, UPLC/QTOF MS analysis was performed using a triple TOF™ 5600 MS/MS system (Sciex, Concord ON, Canada) combined with a UPLC system (Waters Corp., Milford, MS, USA). The UPLC system used to elute and separate polar metabolites was equipped with an Acquity UPLC HSS T3 column (2.1 × 100 mm with 1.7-μm particles; Waters Corp). The column oven temperature was maintained at 40 °C, and the autosampler temperature was maintained at 4 °C. The flow rate was set to 0.45 mL/min for 22 min, and the injection volume was 5 µL.

The binary gradient system comprised LC–MS-grade water with 0.1% formic acid (solvent A) and LC–MS-grade ACN with 0.1% formic acid (solvent B). The linear gradients were as follows: 1% B, 1–10% B from 0 to 5 min, 10–30% B from 5 to 11 min, 30–50% B from 1 to 16 min, 50–70% B from 16 to 17 min, 70%–90% B from 17 to 18 min, 90% B from 18 to 20 min, 90%–1% B from 20 to 21 min, 1% B from 21 to 22 min. Every sample was analyzed in positive and negative ionization modes. The mass range was set at 50–1000 m/z. The other parameter settings were as follows: ion source temperature, 600 °C; ion spray voltage, 5.5 kV (ESI+) or −5.5 kV (ESI−); curtain gas flow, 45 psi; nebulizer gas, 50 psi; heater gas, 60 psi; collision energy, 10 V (ESI+) or −10 V (ESI−).

Metabolites were identified by comparing the experimental data with data in various online databases, namely, the Human Metabolome Database (HMDB; http://hmdb.ca), MASSBANK (http://www.massbank.jp), and METLIN (http://metlin.scripps.edu). Some metabolites were confirmed by comparing their MS/MS patterns and retention time with those of commercial standards.

### 2.6. Statistical Analysis

Spectral data were processed with MarkerView software (AB Sciex, Concord, ON, Canada) to obtain peak features. The spectral data obtained from urine samples were normalized with the median fold change. We selected features with a relative standard deviation (RSD%) less than 20 in quality control (QC) samples to sort the reproducible features.

Multivariate analysis was performed using SIMCA-P software (version 12.0, Umetrics, Umea, Sweden). Principle component analysis (PCA) and partial least squares-discriminant analysis (PLS-DA) were used as classification methods to discriminate between the placebo group and the seapolynol group. A permutation test using 100 random measurements was performed in order to check the validity of the PLS-DA model. To identify significantly changed metabolites after seapolynol intake, a univariate statistical analysis of the data collected before and after seapolynol intake was performed using R studio (version 1.1.463). Pearson correlation analysis was carried out to evaluate the relationship between metabolite changes using SPSS software, version 21.0 (SPSS 21.0; SPSS Inc., Chicago, IL, USA). All data are expressed as mean ± standard deviation of the mean or fold change; *p*-values < 0.05 were considered statistically significant.

## 3. Results

### 3.1. Alteration of Clinical Characteristics after 12 Weeks of Intervention

**Figure 1:**
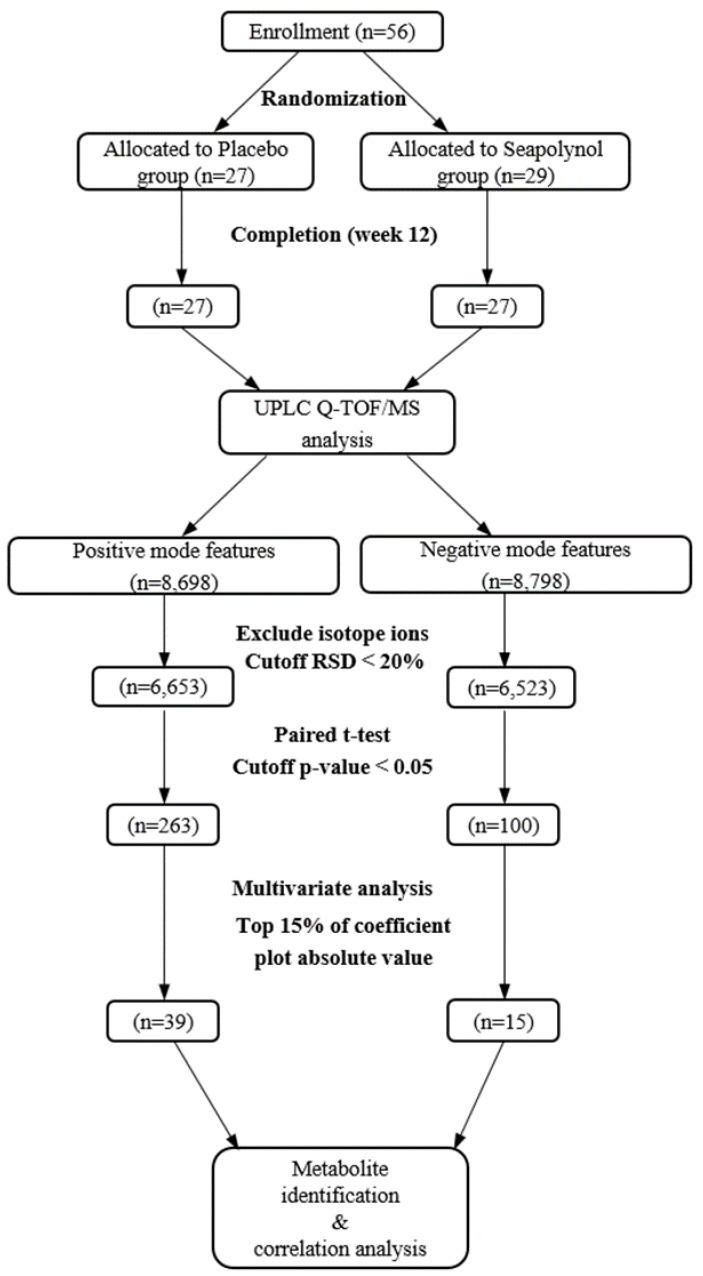
Flow diagram of the study design.

The flow diagram of the study is shown in Figure 1. Among the initial experimental subjects, clinical characteristics were obtained from 56 subjects, and all clinical data used in this analysis are presented (Table 1). The BMIs of the placebo group (*n* = 27) and the seapolynol group (*n* = 27) were 27.53 kg/m^2^ and 27.20 kg/m^2^, respectively, and there were no significant differences between the two groups. The values of clinical characteristics related to body fat, such as percent body fat and fat/lean ratio, were significantly decreased after seapolynol intake, whereas they did not change in the placebo group.

### 3.2. Global Profiling of Human Urine Using UPLC/QTOF MS

**Figure 2:**
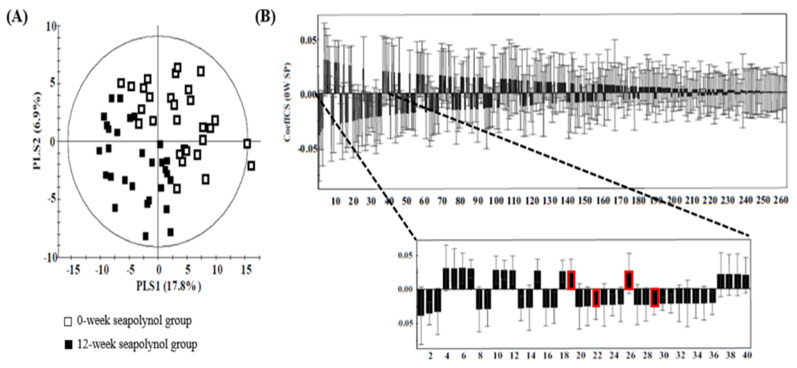
Partial least squares-discriminant analysis (PLS-DA) scatterplot of features that were significantly changed by seapolynol intake in the seapolynol group. (**A**) The PLS-DA scatter plot obtained from UPLC/QTOF MS spectra of human urine samples in positive ionization mode shows that the values at 0 weeks and at 12 weeks for the seapolynol grous are markedly separated (R2x = 0.247, R2y = 0.653, Q2 = 0.273). (**B**) Coefficient plot of the PLS-DA model. Features in the top 15% of the absolute values of the coefficient plot were selected to identify the metabolites significantly changed after seapolynol intake. The red boxes represent the coefficient plot value of the identified metabolites.

Global profiling was conducted using UPLC/QTOF MS for the placebo and seapolynol intake groups. To confirm the stability of the method, QC samples were inserted after every eight samples. As shown in Figure 1. 8698 and 8798 features were obtained from the aligned spectra in positive and negative ionization mode, respectively. Then, isotope ions were excluded, and features were selected with an RSD% less than 20. As a result, 6653 and 6523 spectral features were used in further statistical analysis. Multivariate analysis is useful to compare differences among groups. However, we did not observe metabolic differences between the placebo and the seapolynol groups in the PCA score plots (data not shown). To sort features related to seapolynol intake, a paired t-test was performed to compare the placebo and the seapolynol groups, and differences with *p*-values < 0.05 were selected as indicative of metabolites altered significantly by seapolynol intake. After the exclusion of features overlapping in the two groups, 263 and 100 features were identified in the positive and negative ionization mode, respectively. For these features, the PLS-DA score plot showed a clear difference in the subjects in the seapolynol group between weeks 0 and 12. The first partial least squares (PLS1) values were 17.3% and 24.1% in the positive and negative ion modes, respectively. Each group was clustered in the elliptical region of Hotelling’s T2, defined as the 95% confidence interval (Figure 2A; R2X = 0.247, R2Y = 0.653, Q2 = 0.273 in positive mode), and the PLS-DA model was validated using the permutation test (Supplementary Figure S1). Features in the top 15% of the absolute values of the coefficient plot were selected and then identified as importantly changed polar metabolites after seapolynol intake (Figure 2B).

### 3.3. Identification of Urine Metabolites

**Figure 3:**
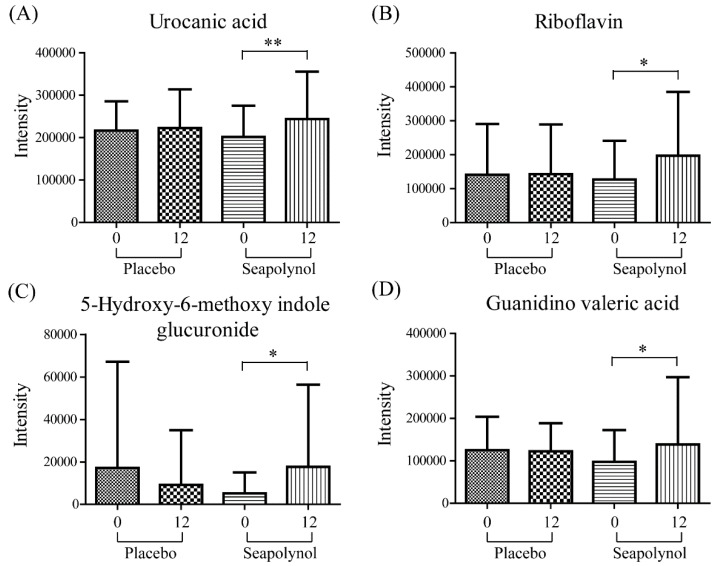
Changes in the levels of the identified metabolites. The levels of (**A**) urocanic acid, (**B**) riboflavin, (**C**) 5-hydroxy-6-methoxyindole glucuronide, and (**D**) guanidino valeric acid were significantly increased over time in the seapolynol group (*p* = 0.004, 0.027, 0.039, and 0.025, respectively). Data are presented as means ± standard deviations.

Thirty-nine and 15 features were identified in the top 15% of the absolute values of the coefficient plots in positive and negative ionization modes, respectively, by comparing the experimental data with data in various online databases, namely, HMDB (http://hmdb.ca), MASSBANK (http://www.massbank.jp), and METLIN (http://metlin.scripps.edu). The selected metabolites are listed in Supplementary Table S1 (positive ionization mode) and Supplementary Table S2 (negative ionization mode). As a result, four metabolites were putatively identified in the positive ionization mode, i.e., riboflavin, urocanic acid, 5-hydroxy-6-methoxyindole glucuronide, and guanidino valeric acid (Figure 3). The identification of metabolites was limited by the lack of databases to identify digested exogenous metabolites [13]. Two of four putatively identified metabolites, riboflavin and urocanic acid, were confirmed using available chemical standards by comparing their MS/MS patterns and retention time (Supplementary Figure S2). In the case of 5-hydroxy-6-methoxyindole glucuronide and guanidino valeric acid, the MS/MS fragment patterns were compared with those of methoxyindole, guanidine, and valeric acid. As shown in Figure 3, riboflavin, urocanic acid, 5-hydroxy-6-methoxyindole glucuronide, and guanidino valeric acid were significantly increased in subjects in the seapolynol group after 12 weeks compared with 0 weeks (*p* = 0.027, 0.004, 0.039, and 0.025, respectively), while these metabolites showed a smaller increase or were not significantly changed in the placebo group over the same time period. Although these identified metabolites had large within-group variations and did not show large differences in the mean before and after seapolynol intake, significant differences were identified in the paired difference test.

To evaluate whether the increased levels of riboflavin resulted from seapolynol, the seapolynol extracts used in this study were directly analyzed using UPLC/QTOF MS. We did not detect riboflavin in the seapolynol extracts. In addition, since both the placebo and the seapolynol intake groups had no specific dietary restrictions, an increase in the riboflavin level could not be considered the result of seapolynol intake.

### 3.4. Correlation between the Levels of Significant Metabolites and Body Fat-Related Clinical Markers

**Figure 4:**
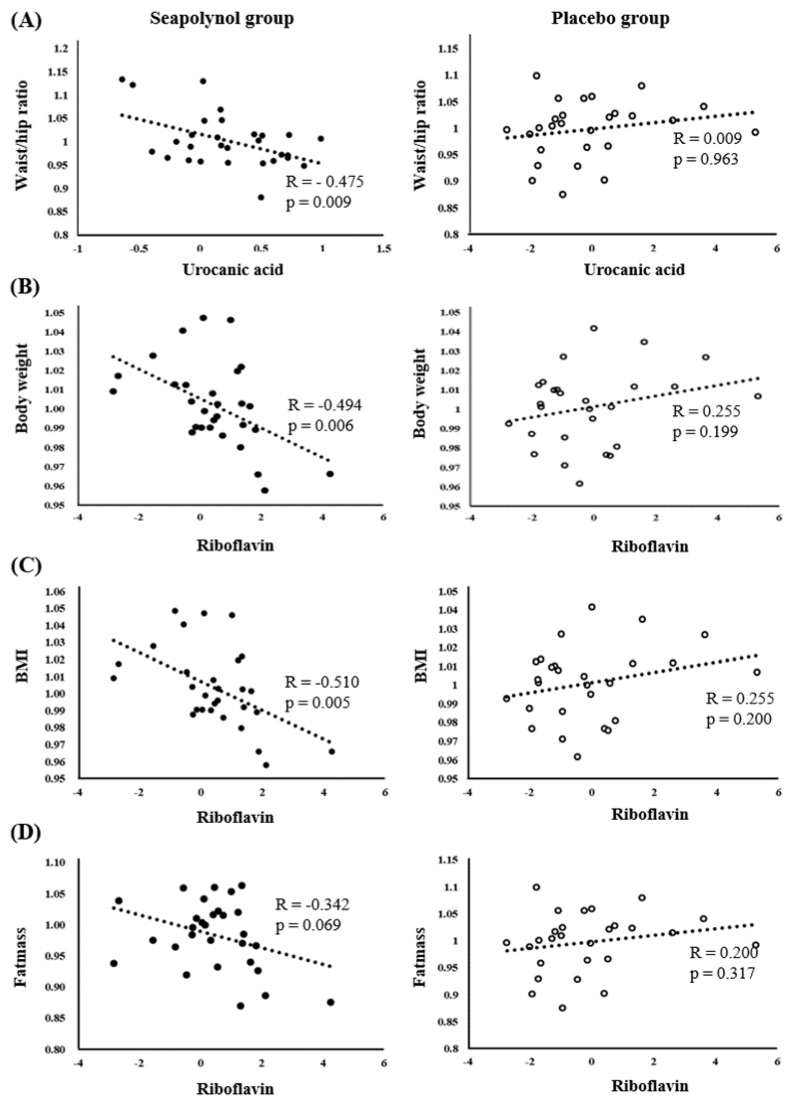
Relationships of riboflavin and urocanic acid with several clinical characteristics related to fat mass in the placebo and seapolynol groups. The x-axis is the log-transformed fold change (12 weeks/0 weeks) in the metabolite level, and the y-axis represents the fold change of the clinical index in each group. Relationship between (**A**) urocanic acid and waist/hip ratio, (**B**) riboflavin and BMI, (**C**) riboflavin and body weight, and (**D**) riboflavin and fat mass. R and p represent correlation coefficient and *p*-value, respectively.

To identify the association between the metabolites changed in the seapolynol intake group and the clinical characteristics related to body fat, a correlation analysis was performed for both the placebo and the seapolynol groups (Table 2). The data used in Pearson correlation analysis were binary log-transformed (log2) fold changes in each metabolite and fold changes in clinical characteristics between seapolynol-intake subjects at 0 weeks and 12 weeks. In the seapolynol group, riboflavin and urocanic acid showed a statistically significant correlation with several clinical characteristics related to body fat. Urocanic acid was negatively correlated with waist/hip ratio (*p* = 0.009, Figure 4A), and riboflavin showed a reverse correlation with body weight, BMI, and fat mass (*p* = 0.006, 0.005 and 0.069, respectively, Figure 4B–D). In contrast, increases in 5-hydroxy-6-mothoxyindole glucuronide and guanidino valeric acid showed no significant positive or negative correlation with clinical characteristics, except for a weak negative relationship with triglycerides (TG).

## 4. Discussion

This study performed a metabolic profiling to investigate the metabolic changes induced by seapolynol intake in healthy human urine samples. To the best of our knowledge, this is the first study to demonstrate the metabolic effect of seapolynol intake in healthy humans. We found that changes in body weight, BMI, and waist/hip ratio were negatively associated with changes in riboflavin and urocanic acid in the seapolynol group (Table 2); in contrast, there were no significant associations between these measurements in the placebo group. Therefore, the decreases in body weight and BMI and waist/hip ratio can be considered to be closely related to the increase in riboflavin and urocanic acid induced by seapolynol intake. Many researchers have tried to clarify the associations between the amount of antioxidants in the human body and clinical characteristics related to obesity. According to these studies, BMI, body weight, waist circumference, and waist/hip ratio were all inversely correlated with the levels of antioxidant compounds such as carotenoid, α-tocopherol, ascorbic acid, and β-carotene in plasma or serum. In particular, BMI was significantly inversely correlated with the levels of most antioxidants [21,22,23,24]. In addition, the antioxidant levels detected in plasma and serum samples from obese populations were lower than those from nonobese populations [25]. Our finding that the increased levels of riboflavin were negatively correlated with BMI is consistent with previous studies and clearly shows that riboflavin can induce body fat reduction.

*E. cava* extracts could be noteworthy in that phlorotannin-rich extracts showed potent peroxynitrite scavenging activities in addition to general antioxidant activities [26]. *E. cava* extracts contain many bioactive polyphenol compounds referred to as phlorotannins, including bioactive secondary metabolites such as dieckol, bieckol derivatives, phlorofurofucoeckol A, and phloroeckol [2]. Several studies have reported that polyphenolic compounds, including phlorotannin derivatives obtained from *E. cava*, have strong antioxidant activity against free radicals in functional food and pharmaceutics [2,27]. Seapolynol, a polyphenol extracted from *E. cava*, has the ability to inhibit weight gain and fat accumulation in a high-fat-diet-induced obese mouse model [28] and to significantly decrease body weight, BMI, body fat ratio, and waist circumference in overweight Korean individuals [3].

Riboflavin is a biological precursor of flavin adenine dinucleotide (FAD) and flavin mononucleotide (FMN), which play an important role as endogenous metabolites in the structure and function of flavin proteins [26,27,29,30,31]. Riboflavin has been reported to affect the oxidation state of the body, such as lipid peroxidation [30]. In particular, the relationship between riboflavin and obesity has been considered for the development of treatments to control obesity. Riboflavin deficiency has been shown to promote inflammation in adipocytes, resulting in an obese state and consequently leading to fatal chronic inflammation [31]. Our data suggest that seapolynol intake may further enhance the antioxidant activity of riboflavin, which is helpful for improving obesity.

Elevated oxidative stress is another factor that contributes to obesity-linked inflammation [32]. Previous studies have shown that the highly hydrophilic phlorotannins of *E. cava* have strong inhibitory effects on free radicals and DNA damage [1]. The level of malondialdehyde, an indicator of lipid peroxidation, was significantly increased in a high-fat diet group compared to the control group but decreased in all groups consuming seapolynol extracts. These results show that seapolynol extracts from *E. cava* can inhibit the overexpression of obesity-induced reactive oxygen species (ROS) [28]. As previous studies reported superior antioxidant activity and obesity reduction as effects of *E. cava* on obesity, obesity treatment by seapolynol intake may be related to riboflavin.

The level of urocanic acid, the deamination product of histidine, has been shown to be elevated in tumor tissue due to the disruption of histamine metabolism or metabolic disorders caused by glutamate [33] and to play a role in improving the degranulation of mast cells in obese mouse models [34]. In our study, the level of urocanic acid was increased after seapolynol intake, and this change was significantly associated with changes in the waist/hip ratio, as shown in Table 2 and Figure 4. Therefore, we expected that an increased level of urocanic acid due to seapolynol intake could improve obesity. However, it is not clear whether urocanic acid has an effect on obesity. Therefore, further studies are needed to investigate how urocanic acid improves obesity.

The compound 5-Hydroxy-6-methoxyindole is a glucuronidation product associated with tryptophan metabolism. According to a study on the effect of obesity improvement by caffeine ingestion, increases in glucuronide-conjugated compounds such as 4-O-glucuronide and 3-indole carboxylic acid glucuronide were observed in a high-fat-diet–caffeine (HFDC) model, induced by caffeine consumption [17]. Guanidino valeric acid is known to be partially degraded to γ-guanidinobutyric acid by chemical oxidation [32]. Fluids can be used as a complementary diagnostic parameter for hyperargininemia. However, we did not observe a significant relationship between the alterations in 5-hydroxy-6-methoxyindole and guanidine valeric acid and the effect of weight loss. This might be because the effect of these compounds on obesity was not significant or because the sample size of our study was small compared to the within-group variation.

Our results were obtained in an initial study with a short follow-up period of 12 weeks. Therefore, the consequences of seapolynol extract intake will continue to be investigated for a longer period. In addition, metabolomics analysis in other biofluids, such as plasma or serum, may be needed later to verify the results in urine

## 5. Conclusions

In this study, we performed metabolomics analysis of human urine to investigate the effect of seapolynol intake. Our metabolomics profiling showed that the levels of metabolites, including riboflavin, urocanic acid, 5-hydroxy-6-methoxyindole glucuronide, and guanidino valeric acid, were changed significantly in the seapolynol intake group compared with the placebo group. In particular, riboflavin was clearly associated with BMI, body weight, fat mass, and percent body fat. This result showed that metabolic profiling based on mass spectrometry could be successfully applied to investigate the metabolic effects of seapolynol intake in humans. This study demonstrates that a metabolomic profiling approach combined with statistical analysis is a useful tool to identify the metabolic changes induced by dietary supplementation with functional foods.

## Figures and Tables

**Table 1:** Clinical characteristics of the study subjects.

Variables	Placebo (*n* = 27)			Seapolynol (*n* = 29)		
	Baseline	After 12 Weeks	*p*-Value	Baseline	After 12 Weeks	*p*-Value
Gender	7/20		-	10/19		-
Age (years)	38.66 ± 11.32		-	36.00 ± 9.9		-
Body weight (kg)	74.12 ± 9.33	74.21 ± 9.62	0.74	76.70 ± 8.24	76.77 ± 7.56	0.83
BMI (kg/m^2^)	27.53 ± 1.62	27.56 ± 1.73	0.79	27.20 ± 1.22	27.28 ± 7.56	0.50
Waist circumference (cm)	89.24 ± 6.91	89.95 ± 8.18	0.46	89.57 ± 6.02	90.71 ± 7.32	0.23
Waist/hip ratio	0.88 ± 0.06	0.88 ± 0.06	0.74	0.88 ± 0.06	0.88 ± 0.06	0.96
Fat mass (kg)	26.59 ± 3.41	26.53 ± 3.80	0.83	26.35 ± 4.09	25.92 ± 4.32	0.11
Percent body fat (%)	35.90 ± 4.74	35.70 ± 4.90	0.52	34.55 ± 6.29	33.88 ± 6.54	0.02
Fat/lean mass ratio	0.57 ± 0.12	0.56 ± 0.12	0.53	0.54 ± 0.14	0.53 ± 0.14	0.02
FMI (kg/m^2^)	9.95 ± 1.42	9.92 ± 1.53	0.83	9.44 ± 1.73	9.31 ± 1.91	0.17
TAT (cm^2^)	405.93 ± 69.02	402.58 ± 75.99	0.61	396.07 ± 72.44	389.17 ± 74.56	0.15
VAT (cm^2^)	125.50 ± 50.14	126.12 ± 57.66	0.89	106.92 ± 35.82	105.56 ± 31.10	0.68
TG (mg/dL)	131.37 ± 101.66	143.96 ± 104.27	0.20	110.00 ± 70.80	114.40 ± 74.81	0.69
TC (mg/dL)	201.52 ± 37.13	200.04 ± 39.96	0.72	193.00 ± 30.20	190.70 ± 27.85	0.51
LDL-C (mg/dL)	132.30 ± 32.06	131.22 ± 35.44	0.78	126.00 ± 24.90	121.30 ± 25.06	0.21

Values represent the mean ± standard deviation. BMI, body mass index; FMI, fat mass index; TAT, total adipose tissue; VAT, visceral adipose tissue; TG, triglyceride; TC, total cholesterol; LDL-C, low density lipoprotein cholesterol.

**Table 2:** Correlation between significantly changed metabolites and clinical characteristics (a, urocanic acid, b, guanidino valeric acid, c, 5-hydroxy-6-methoxyindole glucuronide, d, riboflavin).

Correlation Coefficient r	Body Weight	BMI	Waist/Hip Ratio	Fat Mass	Percent Bodyfat	Fat/Lean Ratio	FMI	TAT	VAT	SAT	TC	LDL
a	−0.117	−0.162	−0.475 ***	0.061	0.135	0.156	0.059	−0.212	−0.149	−0.049	0.347	0.247
b	0.121	0.116	0.081	0.034	−0.050	−0.014	0.033	0.386	0.243	0.189	−0.050	−0.067
c	−0.082	−0.081	0.304	−0.137	−0.156	−0.155	−0.136	0.217	0.106	0.163	−0.230	−0.127
d	−0.494 ***	−0.510 ***	−0.082	−0.342	−0.259	−0.221	−0.343	−0.033	−0.129	0.097	−0.113	−0.123

*** means p-value < 0.001. BMI, body mass index; FMI, fat mass index; TAT, total adipose tissue; VAT, visceral adipose tissue; TG, triglyceride; TC, total cholesterol; LDL-C, low density lipoprotein cholesterol.

## Supplementary Materials

The following are available online at https://www.mdpi.com/2072-6643/12/5/1407/s1, Table S1: Selected metabolites in positive ionization mode, Table S2: Selected metabolites in negative ionization mode, Figure S1: MS/MS spectra acquired from commercial standard compounds (A and C) and plasma samples (B and D). Riboflavin (A and B) and urocanic acid (C and D).
