# Supplementary material for: Urinary Metabolomic Profiling Analysis and Evaluation of the Effect of Ecklonia cava Extract Intake
Source: Nutrients. 2020 May 14;12(5):1407. doi: 10.3390/nu12051407 (PMC7285171; doi:10.3390/nu12051407)

Supplementary Figure 1. Validation plot of the PLS-DA model using a permutation test the was randomly permuted 100 times, and the resulting R2 and Q2 values were plotted.

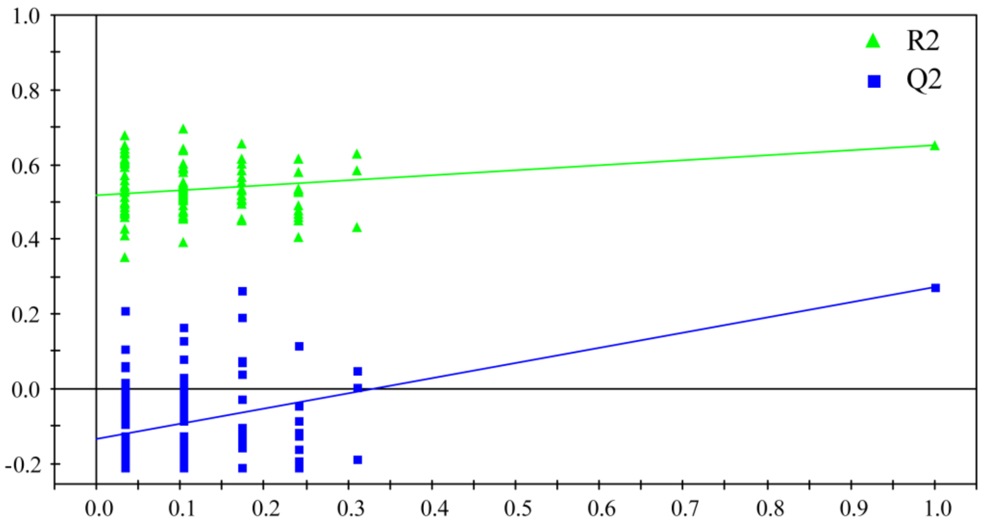

Supplement: Supplementary file 1 [file nutrients-12-01407-s001.zip › Supplementary Materials_nutrients/Supplementary Figure 1.pdf]
