# Supplementary material for: Urinary Metabolomic Profiling Analysis and Evaluation of the Effect of Ecklonia cava Extract Intake
Source: Nutrients. 2020 May 14;12(5):1407. doi: 10.3390/nu12051407 (PMC7285171; doi:10.3390/nu12051407)

Supplementary Figure 2. The MS/MS spectra acquired from commercial standard compounds (A and C) and plasma samples (B and D). Riboflavin (A and B) and urocanic acid (C and D).

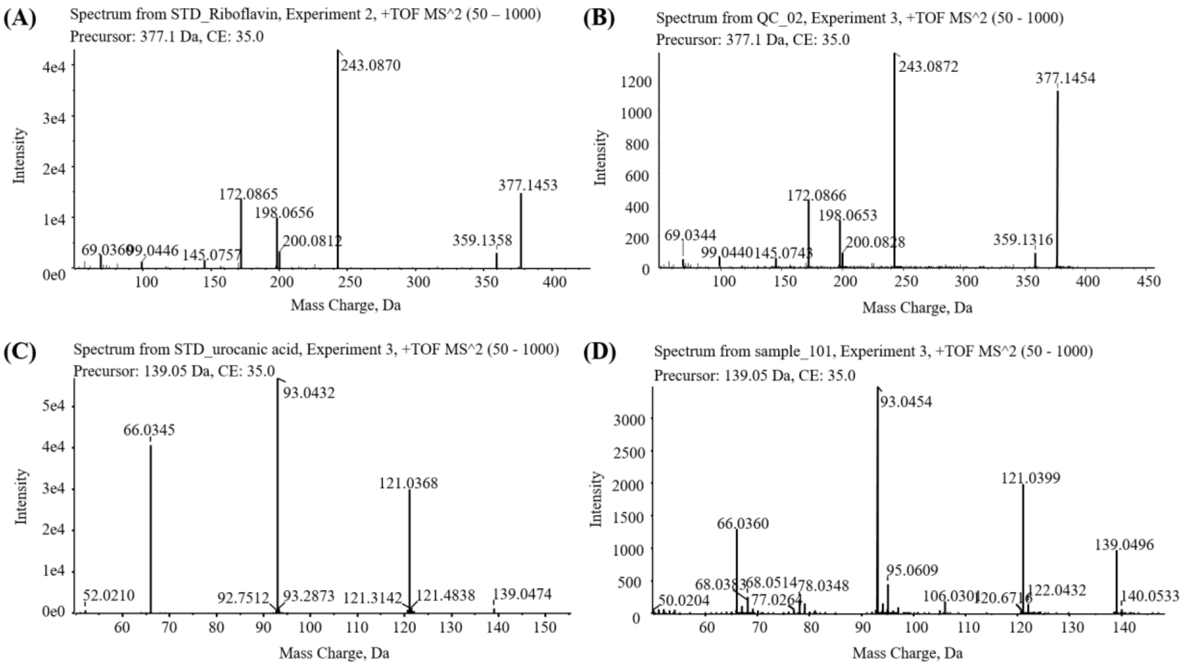

Supplement: Supplementary file 1 [file nutrients-12-01407-s001.zip › Supplementary Materials_nutrients/Supplementary Figure 2.pdf]
